# Somatostatin signalling promotes the differentiation of rod photoreceptors in human pluripotent stem cell‐derived retinal organoid

**DOI:** 10.1111/cpr.13254

**Published:** 2022-05-28

**Authors:** Mingkang Chen, Xiying Mao, Darui Huang, Jiaona Jing, Wenjun Zou, Peiyao Mao, Mengting Xue, Wenjie Yin, Ruiwen Cheng, Yan Gao, Youjin Hu, Songtao Yuan, Qinghuai Liu

**Affiliations:** ^1^ Department of Ophthalmology The First Affiliated Hospital of Nanjing Medical University Nanjing China; ^2^ Department of Ophthalmology The Affiliated Huaian No. 1 People's Hospital of Nanjing Medical University Huaian China; ^3^ Department of Ophthalmology Children's Hospital of Nanjing Medical University Nanjing China; ^4^ Department of Ophthalmology The Affiliated Wuxi No. 2 People's Hospital of Nanjing Medical University Wuxi China; ^5^ Department of Ophthalmology, Shanghai General Hospital Shanghai JiaoTong University School of Medicine Shanghai China; ^6^ Zhongshan Ophthalmic Center, State Key Laboratory of Ophthalmology Sun Yat‐sen University Guangzhou China

## Abstract

**Objectives:**

Stem cell‐derived photoreceptor replacement therapy is a promising strategy for the treatment of retinal degenerative disease. The development of 3D retinal organoids has permitted the production of photoreceptors. However, there is no strategy to enrich a specific photoreceptor subtype due to inadequate knowledge of the molecular mechanism underlying the photoreceptor fate determination. Hence, our aim is to explore the uncharacterized function of somatostatin signalling in human pluripotent stem cell‐derived photoreceptor differentiation.

**Materials and Methods:**

3D retinal organoids were achieved from human embryonic stem cell. The published single‐cell RNA‐sequencing datasets of human retinal development were utilized to further investigate the transcriptional regulation of photoreceptor differentiation. The assays of immunofluorescence staining, lentivirus transfection, real‐time quantitative polymerase chain reaction and western blotting were performed.

**Results:**

We identified that the somatostatin receptor 2 (SSTR2)‐mediated signalling was essential for rod photoreceptor differentiation at the precursor stage. The addition of the cognate ligand somatostatin in human 3D retinal organoids promoted rod photoreceptor differentiation and inhibited cone photoreceptor production. Furthermore, we found that the genesis of rod photoreceptors was modulated by endogenous somatostatin specifically secreted by developing retinal ganglion cells.

**Conclusions:**

Our study identified SSTR2 signalling as a novel extrinsic regulator for rod photoreceptor fate determination in photoreceptor precursors, which expands the repertoire of functional signalling pathways in photoreceptor development and sheds light on the optimization of the photoreceptor enrichment strategy.

## Graphical abstract

The somatostatin receptor 2 (SSTR2)‐mediated signalling modulates the fate determination of photoreceptor precursors in hESC‐derived retinal organoids. The activation of SSTR2 signalling is critical for rod photoreceptor differentiation. Specifically, somatostatin increases rod photoreceptor production, while SSTR2 antagonist promotes the fate bias of photoreceptor precursors towards the cone photoreceptors.

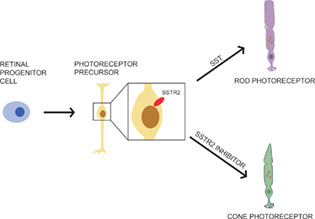

## 1 INTRODUCTION

Photoreceptors are highly specialized sensory neurons in the retina that contribute to visual information. Cone photoreceptor enables colour and high‐acuity vision, while rod photoreceptor mediates vision in the dark.^1^ The dysfunction or death of photoreceptors in the human retina leads to visual impairment in retinal degenerative diseases, a major cause of incurable blindness worldwide.^2^ Photoreceptor replacement is currently under development and aims to provide a promising therapeutic approach for retinal degenerative diseases.^3^ Considerable progress has been made in establishing human pluripotent stem cell‐derived 3D retinal organoids that permit the generation of bonafide photoreceptors.^4^ However, the limited and unpurified photoreceptor population generated in the culture prevents the clinical application of cell‐based therapy.

The establishment of a photoreceptor enrichment strategy is hampered by our inadequate knowledge of the molecular mechanism underlying human photoreceptor development. Photoreceptor development is achieved by the intrinsic regulation of transcriptional factors coordinating with extrinsic signals during retinogenesis.^5^ Photoreceptors are derived from retinal progenitor cells (RPC) which undergo the terminal neurogenic divisions to become photoreceptor precursors. These precursors further differentiate into cones or rods at specified developmental stages. Several key transcription factors are required for photoreceptor commitment, such as orthodenticle homeobox 2 (OTX2), PR/SET domain 1 (PRDM1) and cone‐rod homeobox protein (CRX). These factors simultaneously regulate the expression of both cone and rod photoreceptor‐specific genes.^6^ Neural retina‐specific leucine zipper (NRL), a basic‐motif leucine zipper transcription factor of the Maf subfamily, is known as the earliest marker of rod photoreceptors. However, little is known about the regulatory mechanism of NRL expression in photoreceptor precursors. The expression of NRL can be influenced by extrinsic factors, including positive regulators like insulin‐like growth factor 1 (IGF1), retinoic acid (RA) and taurine.^7^, ^8^ By contrast, ciliary neurotrophic factor (CNTF) and leukaemia inhibitory factor (LIF) negatively regulate NRL and rhodopsin expression and ultimately rod differentiation. However, how these signalling pathways are integrated into the segregation of cone‐ and rod‐specific gene regulatory network remains largely unknown.

In this study, by leveraging the published single‐cell transcriptome datasets of human retinal development, we identified a novel pathway involving the somatostatin receptor 2 (SSTR2) for rod photoreceptor differentiation in the precursor stage.^9^, ^10^ SSTR2, as one of the five somatostatin receptors (SSTR1‐5), regulates the release of several growth hormones.^11^ During development, SSTR2 exhibits dynamic expression patterns in the embryonic brain and exerts multiple functions in neurogenesis. In the differentiation culture of embryonic stem cells (ESCs), SSTR2 facilitates the self‐renewal of ESCs via the activation of the LIF/STAT3 signalling.^12^, ^13^, ^14^ In human retinogenesis, we found that SSTR2 exhibited restricted expression in photoreceptor precursors. Using the human ESC (hESC)‐derived 3D retinal organoid as a model of photoreceptor development, we uncovered that SSTR2‐mediated signalling promoted rod photoreceptor differentiation at the expense of cone photoreceptor production. Furthermore, we revealed that endogenous somatostatin was produced specifically by retinal ganglion cells, which provided a developmental niche for rod photoreceptor differentiation. Taken together, our study helped elucidate a novel mechanism for the cell enrichment strategy of photoreceptor subtypes during photoreceptor replacement therapy.

## 2 MATERIALS AND METHODS

### 2.1 hESC culture

The human embryonic stem cell (hESC) line H9 was kindly provided by the Stem Cell Bank, Chinese Academy of Sciences. Cell culture was conducted in a bio‐safety cabinet using approved biosafety‐level practices. hESCs were expanded in Essential 8 medium (Gibco, Thermo Fisher Scientific, Waltham, MA, USA) in a 6‐well plate precoated with vitronectin (VTN‐N) (Gibco) in 37°C and 5% CO_2_ cell incubator. Versene (Gibco) was used during the passage of the hESCs.

### 2.2 Generation of retinal organoids

The retinal organoids were differentiated following the protocol of a previous study.^15^ When hESCs reached 70% confluency, we used dispase (2 mg/ml, dissolved in DMEM F12, STEMCELL Technologies) to digest the cells at 37°C for 2–3 min until the edge of the cell colonies began to become curly and bright. Subsequently, we gently removed the cells from the vitronectin‐coated plate (Gibco) by a plastic pipette to generate embryoid bodies (EB) and collected all floating colonies by Pasteur pipets into untreated dishes with 7.5 ml Essential 8 medium (Gibco) and 2.5 ml Neural induction medium (NIM: DMEM/F12 containing 1% N2, 1% MEM nonessential amino acids, 1% penicillin–streptomycin, and 2 mg/ml heparin sulfate). For the next seven days, the EBs spontaneously formed a sphere, and the culture was agitated daily along with the replacement of the used medium. After the gradual transition from Essential 8 medium to NIM, EBs were attached to the treated 6‐well plates on Day 7 by adding 2 ml NIM. The day before attachment (Day 6), recombinant human BMP4 (50 ng/ml, R&D Systems) was added to the medium and diluted by changing half the medium every other day, from Day 9 onwards. On Day 16, the attached EBs were lifted by a pipette tip under a microscope and placed in a non‐attachable dish with 10 ml retinal differentiation medium (RDM: DMEM, DMEM F12 [3:1] containing 2% B27 supplement, 1% MEM nonessential amino acids, and 1% penicillin–streptomycin). For long‐term culture, 10% fetal bovine serum, 0.5 μM RA, 100 μM taurine, 2 mM GlutaMAX (Gibco) was supplemented in RDM. Regular trimming and selection of the EBs were needed.

### 2.3 Lentivirus preparation and infection

To suppress the expression of the gene encoding somatostatin (*SST)*, lentivirus carrying shRNA targeting *SST* was designed from VectorBuilder. To perform lentiviral transduction in retinal organoids, 1 million lentiviral transduction units per retinal organoid were added to the medium in the presence of 5 μg/ml polybrene (Sigma‐Aldrich). After incubation for 12 h, lentiviral particles were removed by changing the medium. The lentivirus‐transduced retinal organoids were cultured for 2 to 3 weeks before observation. To assess the efficiency of *SST* knockdown, 661 W cell line, a mouse photoreceptor cell line derived from the retinal tumour, and hESC‐derived retinal organoids were used.^16^ The target sequence for shRNA#1: CCCAACCAGACGGAGAATGAT, shRNA#2: ACGCAAAGCTGGCTGCAAGAA. These sequences are conserved between mouse and human. The 661 W cell line was cultured in DMEM/F12 (Gibco) supplemented with 10% Fetal bovine serum (Gibco) and 1%Penicillin–Streptomycin (Gibco). Quantitative reverse transcriptase PCR (qRT‐PCR) analysis was performed to measure *SST* mRNA expression.

### 2.4 Tissue preparation and immunohistochemistry

Retinal organoids were fixed in 4% paraformaldehyde, sectioned, and stained as previously described.^15^ The working dilutions of the primary antibodies are listed in the Table S1. The secondary antibodies were purchased from Abcam and diluted to 1:1000 in the blocking buffer. The antibodies are listed under Supporting Information.

### 2.5 RNA extraction and qRT‐PCR analysis

The total RNA was extracted with TRIzol (Thermo Fisher Scientific) following the manufacturer's instructions.^17^ The reverse transcription was performed with PrimeScript RT Master Mix (TAKARA BIO). qPCR was performed using ChamQ Universal SYBR qPCR Master Mix (Vazyme). RNA expression of target genes was normalized to *GAPDH*.

### 2.6 Single‐cell RNA‐sequencing (scRNA‐seq) analysis

The sparse data matrices of retinal organoids derived from human pluripotent stem cell lines and human fetal retina were downloaded from the GEO database (GSE138002, GSE142526).^9^, ^10^ All bioinformatic results present in our study are original and have not been published elsewhere. Datasets from different time points were integrated by performing the Canonical Correlation Analysis (CCA) using Seurat (v 3.1). The data were scaled, principal components were computed, nearest neighbours were determined, and Uniform Manifold Approximation and Projection (UMAP) was performed.^18^ Biological clusters were determined by known cell type markers. For Gene Ontology (GO) analysis, Metascape (http://metascape.org/) was used. To find the gene regulatory networks in photoreceptor development, the Single‐Cell Regulatory Network Inference and Clustering (SCENIC) suite was applied to perform regulatory network inference and clustering analysis following the SCENIC tutorial.^19^ Gene co‐expression network analysis was visualized via Cytoscape 3.7.1.^20^

### 2.7 Statistical analyses

All experiments were performed at least three times independently. At least 5 retinal organoids were used in each treatment group. For each organoid per staining, at least 3 images were processed. The results are presented as mean ± SD. The immunohistochemical quantification analysis was performed in Photoshop and ImageJ. The percentage of marker‐positive cells in the neuroblast layer were calculated. Specifically, in each image, the total number of marker‐positive cells was counted and divided per the total number of cells (labelled with DAPI) in the neuroblast layer. All statistical tests were analysed using Prism (GraphPad, USA). Statistical significance was tested using One‐way ANOVA (Tukey corrected multiple comparison test). *p < 0.05, **p < 0.01.

## 3 RESULTS

### 3.1 Recapitulation of photoreceptor differentiation in 3D retinal organoids

**FIGURE 1:**
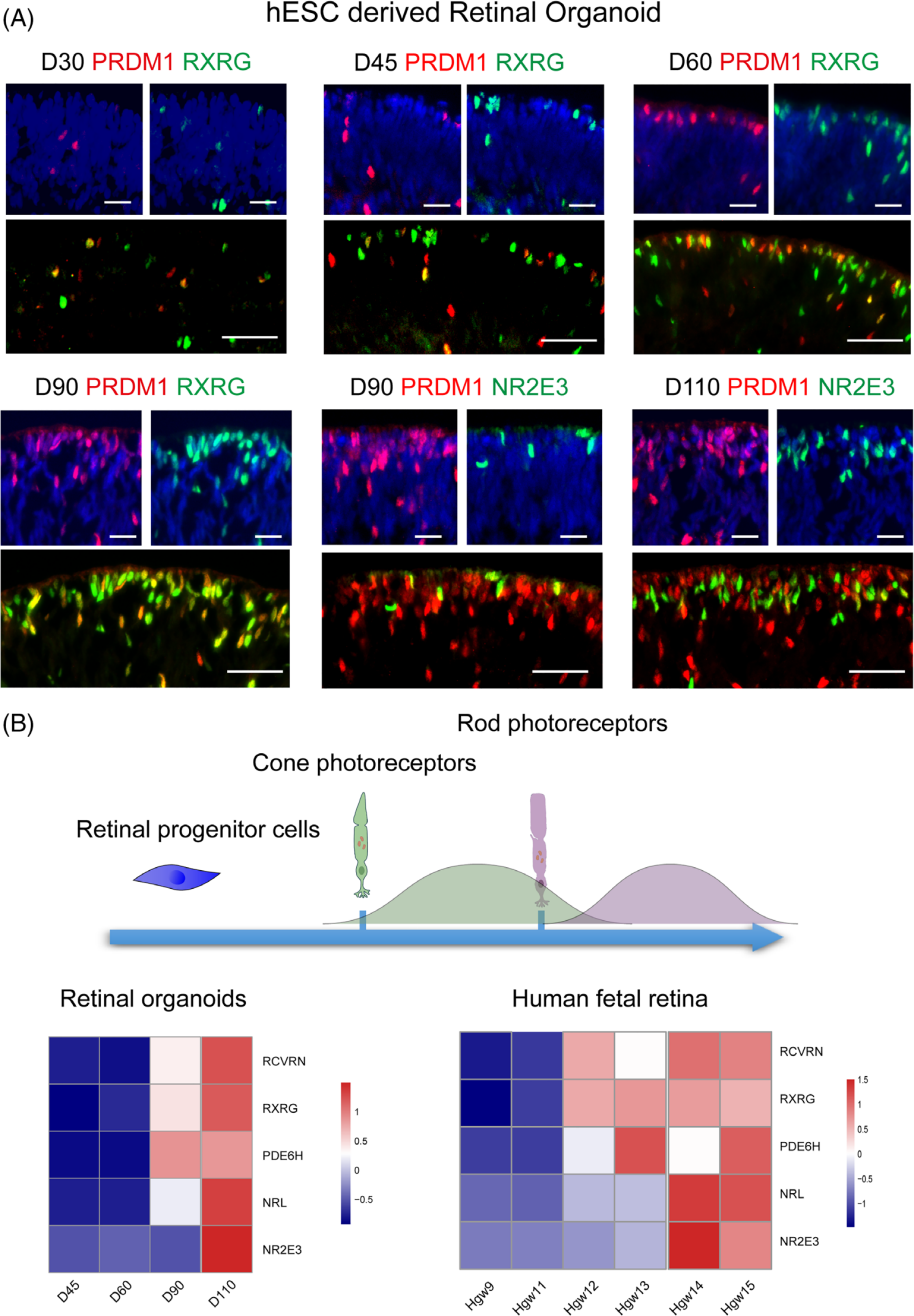
Development of photoreceptors in retinal organoids. (A) Double staining of PRDM1 (red) with RXRG (green) or NR2E3 (green) in retinal organoids at different time points. Scale bar = 50 μm. (B) Schematic diagram showing the development of photoreceptors in retinal organoids. Single‐cell RNA‐sequencing data analysis showing the expression pattern of photoreceptor markers from Day 45 to Day 110 and from Hgw 9 to 15, respectively

Previous studies have reported that the differentiation of retinal organoids recapitulates human retinal development.^10^ To investigate the mechanism of human photoreceptor differentiation, we established the in vitro model of human retinal development based on hESC‐derived 3D retinal organoids (Figure S1A). PRDM1 was previously identified as a photoreceptor marker gene that ubiquitously expressed throughout photoreceptor development, spanning from RPC commitment to terminal differentiation.^21^, ^22^ At Day 30, PRDM1‐positive cells were firstly detected at the outer nuclear layer and co‐expressed with Retinoid X Receptor Gamma (RXRG), indicating the emergence of cone photoreceptor. The number of RXRG‐positive photoreceptors gradually increased during differentiation. At the later stage of differentiation, the PRDM1‐positive population continued to expand, and NR2E3‐positive rod photoreceptors started to emerge around Day 90 and increased over time (Figure 1A). In consistent with the immunofluorescent findings, these marker genes exhibited similar expression patterns at defined developmental stages in transcriptomic data of retinal organoids and largely mirrored their expression in human fetal retina (Figure 1B). Collectively, these results validated the in‐vitro system as a reliable platform for dissecting human photoreceptor development.

### 3.2 Specific expression of SSTR2 in photoreceptor precursors

**FIGURE 2:**
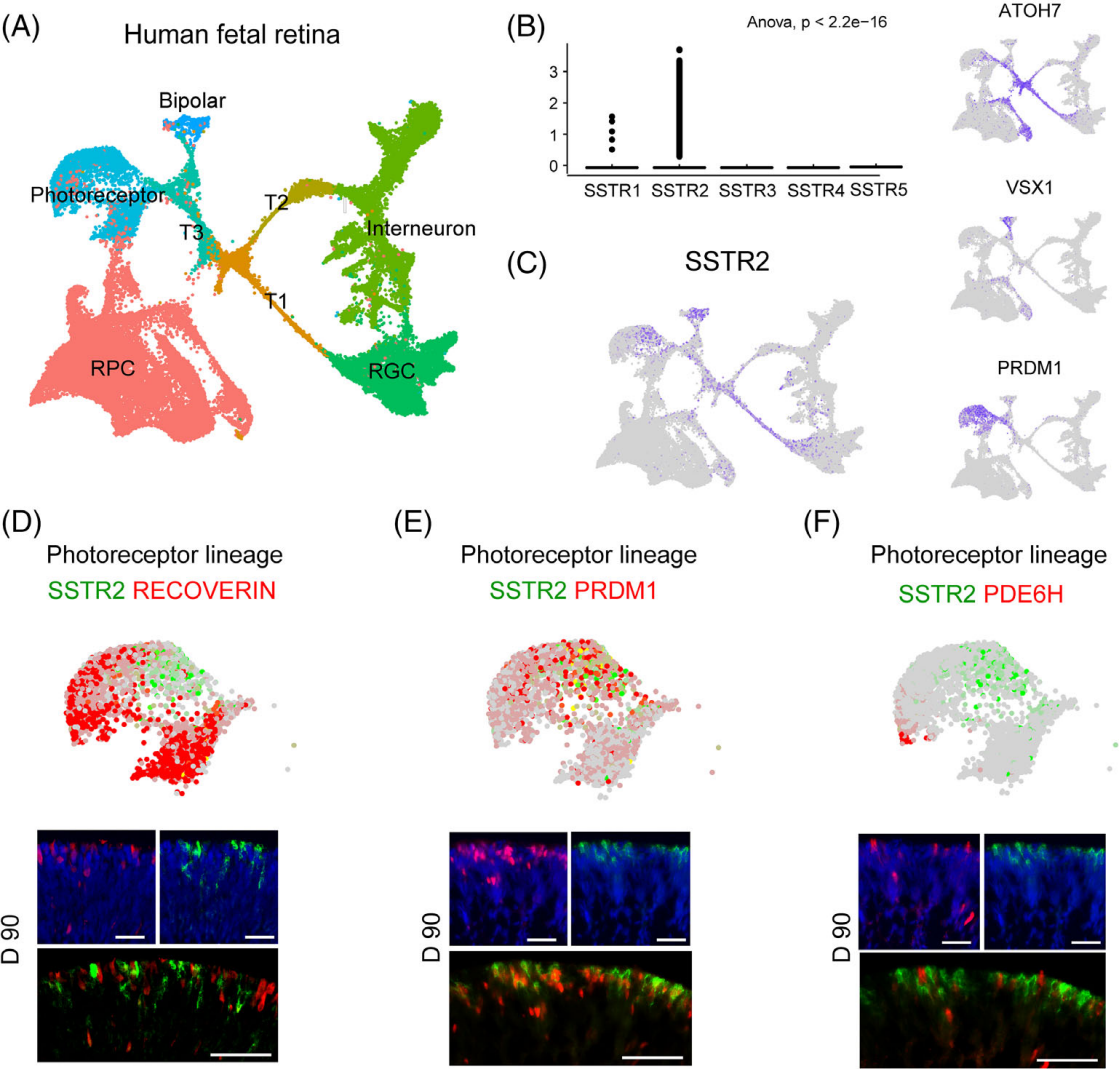
Specific expression of SSTR2 in photoreceptor precursors. (A) UMAP plot of the human fetal retina from Hgw 9 to 16. The clusters were plotted by retinal cell types: photoreceptor cell, bipolar cell, retinal progenitor cell (RPC), interneuron, retinal ganglion cell (RGC), and transition states 1–3 (T1–3). (B) Violin plot showing the expression of somatostatin receptors in the human fetal retina. (C) Distribution of *SSTR2*, *ATOH7*, *VSX1*, and *PRDM1* expression in UMAP plot. (D) No co‐staining is observed for the mature photoreceptor marker RCVRN (red) and SSTR2 (green) in the photoreceptor cluster. Scale bar = 50 μm. (E) The majority of SSTR2 (green) positive cells co‐expressed with photoreceptor precursor marker PRDM1 (red) in the photoreceptor cluster. Scale bar = 50 μm. (F) Mature cone photoreceptor marker PDE6H (red) and SSTR2 (green) show no co‐staining in the photoreceptor cluster. Scale bar = 50 μm

To investigate the expression of somatostatin receptors during retinal development, we used the recently published scRNA‐seq data of human fetal retina across gestation week (Hgw) 9 to 16.^9^ Using unbiased clustering, eight biological clusters were identified, including RPC, retinal ganglion cell (RGC), bipolar cell, interneuron, photoreceptor, and three transitional states (T1, T2, and T3) (Figure 2A). During retinal development, *SSTR2* was the dominant receptor among the somatostatin receptor family (Figure 2B).

To further dissect the spatial expression of SSTR2 in the developing retina, we profiled the expression of *SSTR2* on UMAP plotting. We observed the specific expression of *SSTR2* in the T1 transitional state, bipolar cells, and photoreceptor lineage, marked by atonal homologue 7 (*ATOH7*), visual system homeobox 1 (*VSX1*), and *PRDM1*, respectively^23^, ^24^ (Figure 2C). To focus on the expression of SSTR2 in the photoreceptor lineage, we checked the transcriptome data and confirmed its consistency with the immunofluorescence images. The expression pattern of SSTR2 dynamically changed during the differentiation of retinal organoids (Figure S1B). The majority of SSTR2^+^ cells co‐expressed with PRDM1 and rarely expressed the mature pan‐photoreceptor marker RCVRN in Day 90 retinal organoids (Figure 2D,E). In addition, we observed no overlap between phosphodiesterase 6H (PDE6H) and SSTR2 (Figure 2F). Together, these results implied the specific labeling of SSTR2 in photoreceptor precursors.

### 3.3 SSTR2‐mediated promotion of rod photoreceptor differentiation

**FIGURE 3:**
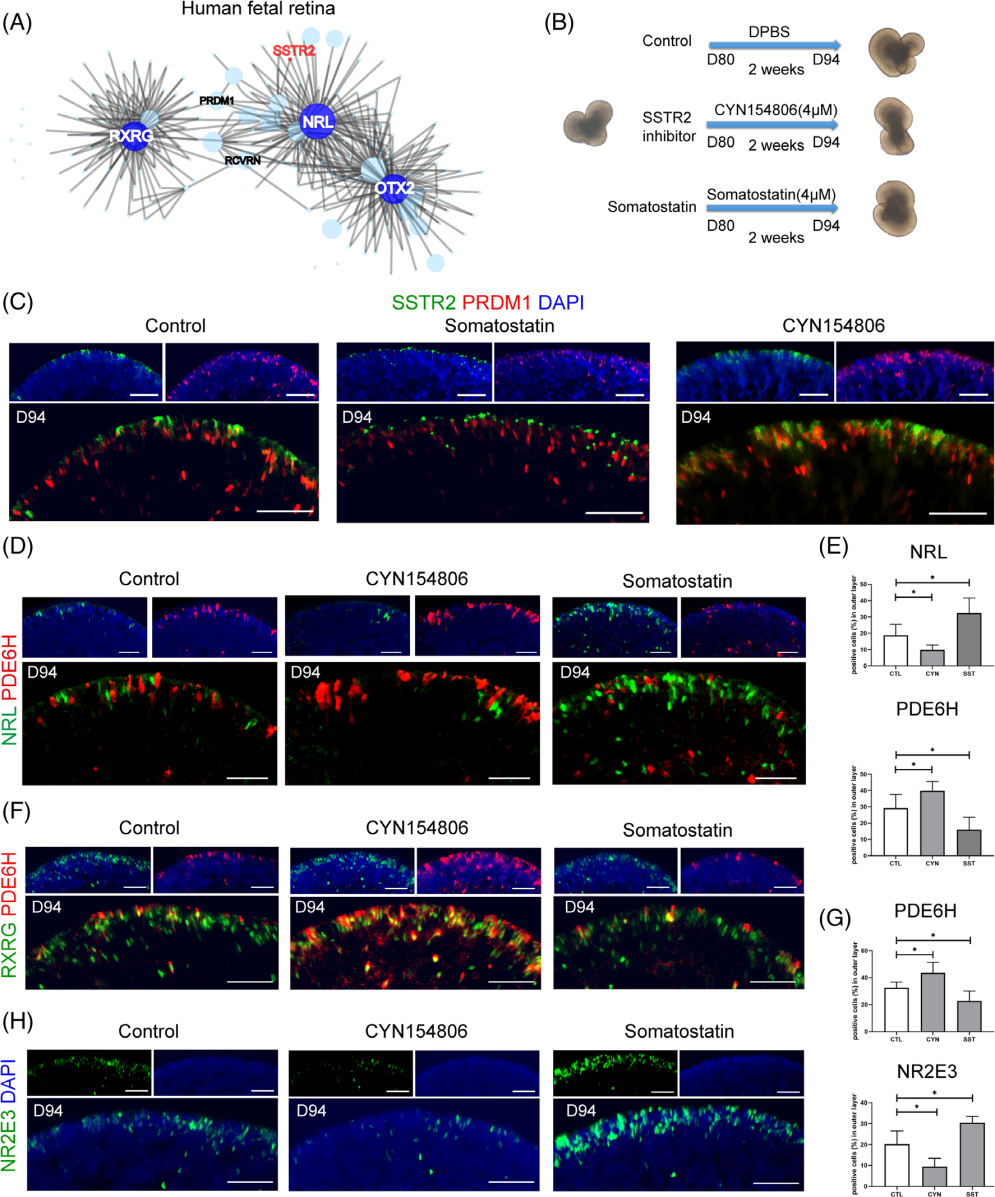
SSTR2‐mediated somatostatin signalling regulates fate switch between cone and rod photoreceptor. (A) The transcriptional co‐expression network of developing photoreceptor lineage in the human fetal retina showing the close connection between *SSTR2* and *NRL*. (B) Schematic diagram showing treatment details of SSTR2 ligand somatostatin and its inhibitor CYN154806. (C) Representative images of activated and internalized SSTR2 (green) co‐stained with PRDM1 (red) in control and somatostatin (SST) and CYN154806 treatment (*n* = 6). Scale bar = 50 μm. (D) The immunostaining of NRL and PDE6H expression. (E) Immunohistochemical quantification analysis of cone photoreceptor marker PDE6H and rod photoreceptor precursor marker NRL. *p < 0.05. (F) Cone photoreceptors labelled by RXRG (green) and PDE6H (red) show a significant increase in CYN154806‐treated organoids (*n* = 4) and a decrease in SST‐treated organoids (*n* = 5). Scale bar = 50 μm. (G) Immunohistochemical quantification analysis of cone photoreceptor marker PDE6H and rod photoreceptor precursor marker NR2E3. *p < 0.05. Scale bar = 50 μm. (H) Immunofluorescence staining of rod photoreceptor marker NR2E3 (green) in somatostatin (SST) and SSTR2 inhibitor CYN154806 treatment

To investigate the specific function of SSTR2 in photoreceptor precursors, we performed SCENIC to reconstruct gene regulatory network activities of developing photoreceptor lineage in human fetal retina (Figure 3A). The gene co‐expression network showed that *SSTR2* was located within the *NRL*‐centered network, indicating the potential effect of SSTR2‐mediated signalling on rod photoreceptor differentiation. Further, the transcriptional factors (TFs) upstream of *NRL* via SCENIC pipeline were revealed, including *FOS*, *JUNB*, *FOXO3*, *CRX* and *RARA*. These TFs were identified as the putative TFs of NRL whose binding motif were over‐represented in the search space around the transcription start site of *NRL* and correlated with the expression of *NRL* in scRNA‐seq data (Figure S2A, Table 1).

**TABLE 1:** Putative TFs of NRL expression revealed by SCENIC pipeline

TF	gene	highConfAnnot	nMotifs	bestMotif	NES	motifDb	coexModule	spearCor	CoexWeight
JUNB	NRL	TRUE	80	transfac_pro__M08921	5.28	10 kb	top3sd	0.329302	0.00903612
FOS	NRL	TRUE	33	dbcorrdb__FOS__ENCSR000EYZ_1__m1	3.3	10 kb	w0.005	0.30167	0.00976795
CRX	NRL	TRUE	8	taipale_cyt_meth__CRX_NTAATCCN_FL	4.81	10 kb	top1sd	0.273863	0.00836799
RARA	NRL	TRUE	4	taipale_cyt_meth__RARA_NRGGTCANNRGGTCAN_eDBD	3.1	10 kb	top1sd	0.267143	0.00220093
FOXO3	NRL	TRUE	33	hocomoco__FOXO3_MOUSE.H11MO.0.B	4.15	10 kb	w0.005Andtop50	0.262975	0.00536981
ATF4	NRL	FALSE	1	yetfasco__YER045C_8	3.06	10 kb	top50perTarget	0.184148	0.00458646
BCLAF1	NRL	FALSE	4	hocomoco__GABPA_HUMAN.H11MO.0.A	4.09	10 kb	w0.001	0.215361	0.00136625
EZH2	NRL	FALSE	1	dbcorrdb__YY1__ENCSR000BKJ_1__m1	3.06	10 kb	w0.001	0.237286	0.00162493
KDM5B	NRL	FALSE	2	dbcorrdb__YY1__ENCSR000BKU_1__m1	4.1	10 kb	top1sd	0.24938	0.00458371
NEUROD1	NRL	FALSE	1	taipale_cyt_meth__ATOH7_ANCATATGNY_eDBD	3.07	10 kb	top3sd	0.380296	0.02625804
RAD21	NRL	FALSE	5	dbcorrdb__POLR3G__ENCSR000EHQ_1__m4	3.2	10 kb	top1sd	0.25129	0.00459551
SREBF1	NRL	FALSE	1	dbcorrdb__SP1__ENCSR000BKO_1__m1	3.16	500 bp	w0.001	0.275158	0.00335473

We then treated Day 80 retinal organoids with somatostatin and SSTR2‐specific inhibitor CYN154806 for two weeks and harvested at Day 94 (Figure 3B). The distribution of SSTR2 expression was internalized by somatostatin treatment, which marked the activation of the receptor^25^ (Figure 3C). The treatment of CYN154806 significantly increased the number of cells that expressed PDE6H, while somatostatin strongly upregulated the expression of NRL and NR2E3 (Figure 3D–H). The production of rod photoreceptors was further boosted after a prolonged somatostatin treatment when harvested at Day 130 (Figure S3A,B). However, the treatment of CYN154806 upregulated the expression of SSTR2, indicating a negative feed‐forward regulation of CYN154806 on SSTR2 expression (Figure 3C). Hence, SSTR2 signalling in photoreceptor precursors promoted the differentiation of rod photoreceptors.

**FIGURE 4:**
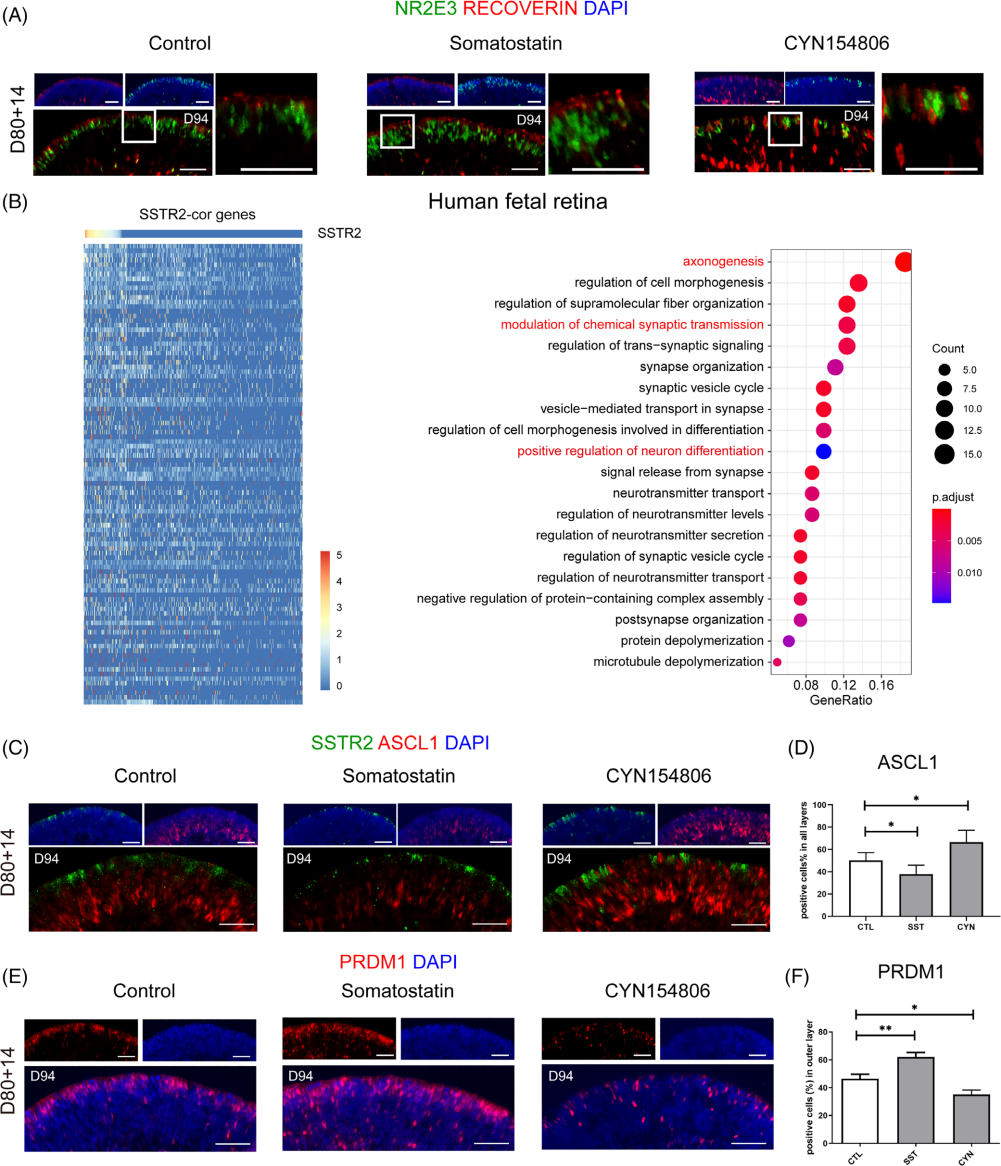
Somatostatin signalling activation promoted photoreceptor maturation. (A) The representative images of RCVRN expression in control and SST treatment. Higher magnification demonstrated the outer and inner segment‐like structure with the treatment of SST. (B) Heatmap showing the genes that positively correlated with SSTR2 by Pearson correlation analysis. GO analysis showing GO terms such as “axonogenesis” and “synaptic transmission” were enriched in the top 100 correlated genes. (C) Representative examples of ASCL1 immunoreactivity (red) show increased expression upon CYN154806 treatment and decreased expression upon SST treatment. (D) Quantification analysis of ASCL1 expression. (E) The increasing expression of PRDM1 in somatostatin treatment and decreasing expression in CYN154806 treatment. (F) Quantification analysis of PRDM1 expression. (*p < 0.05, Scale bar = 50 μm, *n* = 6)

Moreover, the expression of RCVRN, which targets the outer and inner segment‐like structure at the periphery of organoids,^26^ was significantly upregulated in somatostatin‐treated retinal organoids than in the CYN154806‐treated and control organoids (Figure 4A). Consistently, the intersection of the top 100 genes that positively correlated with *SSTR2* in the human fetal retina dataset led to the identification of genes enriched for “axonogenesis” and “synaptic transmission” (Figure 4B). Hence, these results revealed that somatostatin promotes the maturation of the rod photoreceptor.

Interestingly, the expression of the RPC marker Achaete‐Scute Family BHLH Transcription Factor 1 (ASCL1) was greatly downregulated upon somatostatin treatment and upregulated in response to CYN154806 in retinal organoids harvested in Day 94 (Figure 4C,D). ASCL1 is a proneural gene that maintains the proliferative property of RPC.^27^ Furthermore, the differentiation of photoreceptors was enhanced by somatostatin treatment, as evidenced by PRDM1 immunostaining (Figure 4E,F). SSTR2 was previously demonstrated as a tumour suppressor that inhibits cell proliferation. These results suggested that the antiproliferative property of SSTR2 signalling promotes the exit of the cell cycle and the differentiation of neurogenic RPC to photoreceptors.

### 3.4 Endogenous somatostatin in developing retina participates in photoreceptor fate determination

**FIGURE 5:**
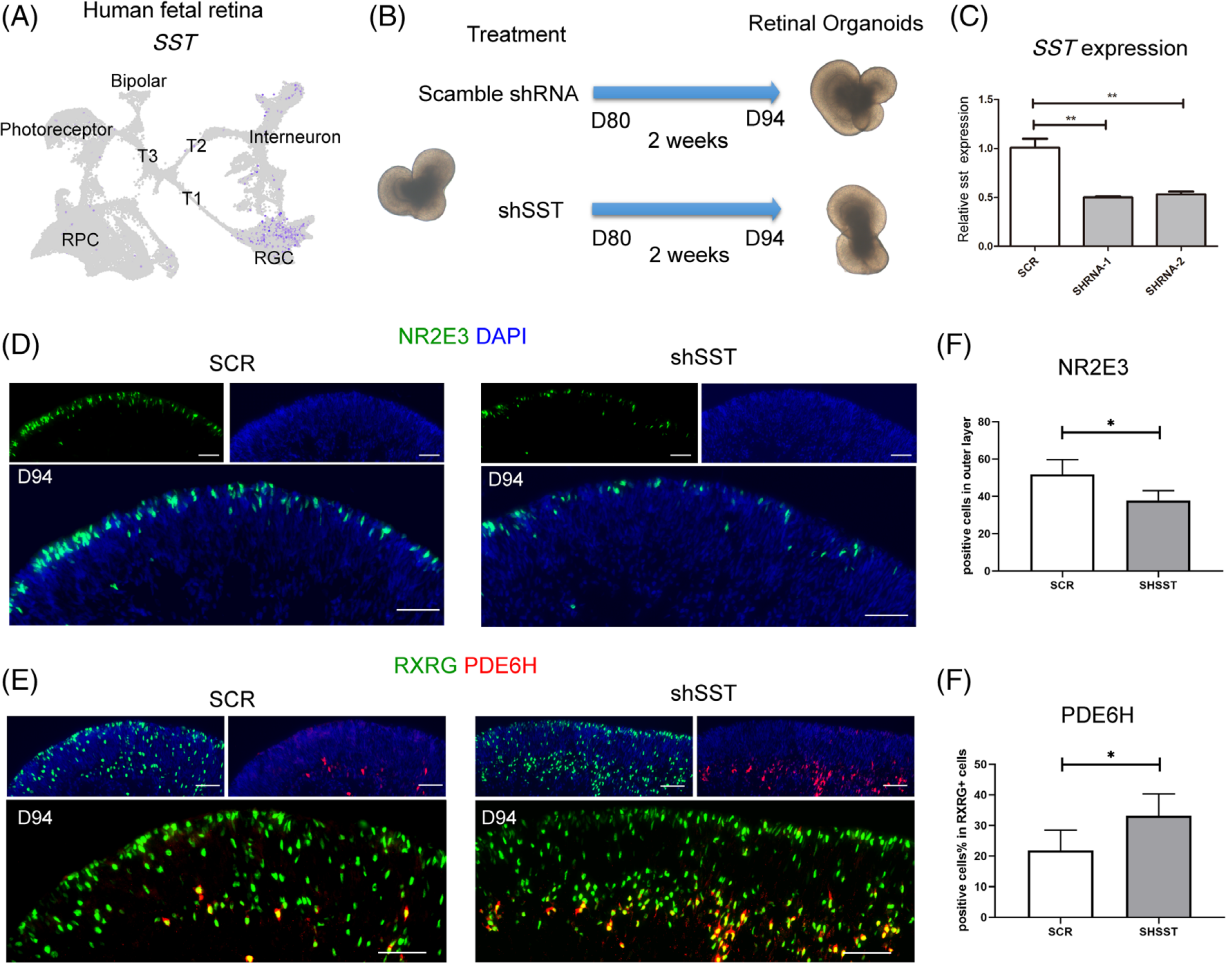
Endogenous somatostatin participates in the fate determination of photoreceptor precursor. (A) UMAP plot showing the restricted expression of *SST* in RGC. (B) Schematic diagram showing the retinal organoids transduced with shSST lentivirus at Day 80 and harvested for observation after two weeks. (C) Quantification of *SST* expression level showing the knockdown efficiency by qPCR. **p < 0.01. (D,E) Immunohistochemical images show reduced rod photoreceptors marked by NR2E3 and increased cone photoreceptors stained with RXRG and PDE6H after knocking down somatostatin. Scale bar = 50 μm. (F) Quantification of NR2E3^+^ cells revealed a significant decrease in the shSST group compared to scramble (SCR). *p < 0.05. (G) Quantification of PDE6H^+^ cells revealed a significant increase in the shSST group compared to the SCR group. *p < 0.05

To investigate the expression pattern of the endogenous somatostatin in the developing retina, we analysed the expression of *SST* in scRNA‐seq data of both human fetal retina and retinal organoid harvested at Day 80. Interestingly, the expression of *SST* was distributed specifically in the cluster of RGC (Figures 5A and S4A,B), which implicated the paracrine effect of RGC on photoreceptor differentiation.

To validate the function of endogenous somatostatin in rod photoreceptor differentiation, we performed a knockdown experiment using lentiviral vectors expressing scramble or *SST*‐targeting shRNA together with mCherry (Figures 5B–C and S4C). Two weeks after lentivirus transduction in D80 retinal organoids, the knockdown of *SST* significantly reduced the number of rod photoreceptors and promoted cone photoreceptor production, as evident from the expression of NR2E3 and PDE6H (Figure 5D–G). Accordingly, CYN154806 treatment further promoted the effects of *SST*‐knockdown on photoreceptor differentiation (Figure S4D). Taken together, these results suggested that somatostatin is a novel extrinsic regulator of fate determination of human retinal photoreceptors.

## 4 DISCUSSION

In this study, we demonstrated that SSTR2‐mediated signalling promotes the generation of rod photoreceptors in retinal organoids. Somatostatin significantly boosted rod photoreceptor production in human retinal organoids, expanding the reservoir of extrinsic stimuli for the direct differentiation of rod photoreceptors.

Somatostatin mediates its effect by activating five transmembrane G‐protein‐coupled receptors (SSTR1‐R5). These receptors have distinct expressional patterns in the retina.^28^ However, in the developing retina in humans, SSTR2 was the only expressed somatostatin receptor specifically distributed in the cluster of photoreceptor precursors, as shown by the transcriptome data and immunostaining. SSTR2 inhibits cell proliferation, serving as a tumour suppressor in non‐neuronal cells.^29^, ^30^ Similarly, we found that SSTR2 signalling significantly downregulated the number of cycling RPCs in retinal organoids, which promoted the differentiation of RPCs to the photoreceptor lineage. Further, the fate of the photoreceptor precursor is biased towards the cone or rod photoreceptors at a given stage.^31^ The activation of SSTR2 signalling in the photoreceptor precursor facilitated rod photoreceptor differentiation, which inhibited the production of the cone photoreceptors.

Somatostatin acts by affecting multiple intracellular signalling pathways.^32^ Protein kinase C (PKC) is the canonical signalling transducer of SSTR, which subsequently activates the Src homology region 2 domain‐containing phosphatase 1 (SHP‐1).^33^ In the embryonic retina, PKC isoform induces rod photoreceptor differentiation, which can be sufficiently abolished by SHP‐1/2 inhibitor.^34^, ^35^ It is hypothesized that SSTR2 signalling regulates rod photoreceptor differentiation via the PKC/SHP pathway. However, how this signalling pathway initiates the gene regulatory network of rod photoreceptors requires further investigation.

In addition, SSTR mediates inhibitory effects on several signalling molecules, such as adenylyl cyclase/adenosine 3′,5′‐cyclic monophosphate, and phosphatidylinositol 3‐kinase (PI3K)/protein kinase B (AKT).^36^ In embryonic retina, AKT phosphorylation decreases in response to IGF1 treatment, providing a favourable niche for rod photoreceptor differentiation.^37^ In a recent study, elevated levels of the PI3K/AKT pathway components were found in the retinal organoid of the retinoblastoma model characterized by cone photoreceptor hyperplasia.^38^ These observations suggested that the cone photoreceptor differentiation is facilitated by an enhanced AKT pathway. We also found that the level of pAKT was significantly upregulated upon treatment with an SSTR2 antagonist (Figure S5A). Therefore, SSTR2 signalling impedes cone photoreceptor differentiation, possibly via the negative regulation of AKT.

Next important question is, how signalling pathway is integrated in the transcriptional hierarchy. Several TFs that putatively target NRL were identified via SCENIC pipeline. RARA is the nuclear receptor for retinoic acid signalling. Retinoic acid is demonstrated to promote rod photoreceptor differentiation.^8^, ^39^, ^40^ CRX also participated in the transcriptional regulation of NRL in cooperation with other factors that direct regulation of distinct gene networks in photoreceptor precursors.^41^, ^42^, ^43^ However, the function of FOS, JUNB and FOXO3 in rod differentiation remains uncharacterized. The expression of FOS and JUNB was PKC/CREB‐dependent, which is modulated by a wide variety of extracellular stimuli, such as growth factors.^44^, ^45^, ^46^ FOXO, on the other hand, was shown to mediate a repressive response of PI3K/AKT pathway.^47^, ^48^, ^49^ Consistently, somatostatin signalling was also demonstrated to activate PKC and repress AKT. Therefore, it indicates the involvement of these TFs in somatostatin‐induced rod differentiation, which awaits further validation.

Further, we illustrated that the knockdown of the endogenous somatostatin expression closely mirrored the phenotype of CYN154806 treatment, suggesting somatostatin signalling as an extrinsic regulator in photoreceptor differentiation. The expression of somatostatin (encoded by *SST*) was high in RGCs. RGC emerged during the first wave of retinal development, way ahead of the genesis of the rod photoreceptors. Previous studies on RGC ablation showed that RPCs at P0 gave birth to the late‐born retinal cells (e.g., rods) and were greatly affected in both *Math5/Brn3b* double null and *Math5* null retinas. In addition, under dark‐adapted conditions, the a‐wave was reduced in the *Math5/Brn3b*‐deficient mice, which was indicative of rod photoreceptor activity.^50^ However, partial loss of RGC resulting from *Brn3b*‐expressing cell ablation or *Math5*‐knockout retinas did not lead to retinal disorganization.^51^, ^52^, ^53^ These studies further suggested the essential function of RGC in the development of other retinal neurons, especially rod photoreceptors.

In summary, we identified SSTR2 signalling as a novel extrinsic regulator for rod photoreceptor fate determination in photoreceptor precursors. The addition of somatostatin significantly increased rod photoreceptor production in human retinal organoid culture, which facilitated the development of a direct differentiation strategy for photoreceptor enrichment. The discovery of SSTR2 signalling in photoreceptor differentiation will expand our understanding of retinal cell fate determination and help improve retinal organoid culture in clinical applications.

## AUTHOR CONTRIBUTIONS

Mingkang Chen, Xiying Mao, Songtao Yuan, Qinghuai Liu were involved in the study design. Songtao Yuan, Qinghuai Liu contributed to materials and reagents and financial support. Mingkang Chen, Xiying Mao, Darui Huang, Jiaona Jing, Wenjun Zou, Peiyao Mao, Mengting Xue, Wenjie Yin, Ruiwen Cheng, Yan Gao performed experiments. Mingkang Chen, Xiying Mao and Qinghuai Liu and Youjin Hu analyzed and interpreted the data. Mingkang Chen, Xiying Mao, Songtao Yuan, Qinghuai Liu drafted the article. All authors have approved the final version of the submitted manuscript.

## CONFLICT OF INTEREST

The authors declared no potential conflicts of interest.

## Supporting information


Appendix S1

## Data Availability

The data are available on reasonable request.
