# Supplementary material for: Somatostatin signalling promotes the differentiation of rod photoreceptors in human pluripotent stem cell‐derived retinal organoid
Source: Cell Prolif. 2022 May 28;55(7):e13254. doi: 10.1111/cpr.13254 (PMC9251046; doi:10.1111/cpr.13254)
Supplement: Supplementary file 1 — Appendix S1 [file CPR-55-e13254-s001.docx]

**SUPPLEMENTARY MATERIALS**

Supplementary Table 1: Antibodies information including sources and identifiers.

| **ANTIBODY** | **SOURCE** | **IDENTIFIER** |
| --- | --- | --- |
| PRDM1 | Santa cruz | Cat# sc-47732 |
| RXRG | Santa cruz | Cat# sc-365252 |
| NR2E3 | Novus | Cat# PP-H7223-00 |
| RECOVERIN | Millipore | Cat# AB5585 |
| PDE6H | Atlas | Cat# HPA045118 |
| ISLET1 | Abcam | Cat# ab109517 |
| mCherry | Biorbyt | Cat# orb182397 |
| ASCL1 | ABCAM | Cat# AB211327 |
| p-AKT | Cell Signaling echnology | Cat# 4060T |
| SST | Santa cruz | sc-55565 |
| Beta actin | Servicebio | GN110011 |
| SSTR2 | Santa cruz | Cat#sc-365502 |
| Donkey anti-mouse 488 | Abcam | Cat# AB50109 |
| Donkey anti-rat 594 | Abcam | Cat# AB150156 |
| Donkey anti-mouse 594 | Abcam | Cat# AB150112 |
| Donkey anti-rabbit 647 | Abcam | Cat# AB150063 |
| Donkey anti-goat 568 | Abcam | Cat# AB175704 |

**Figure S1. The expression patterns of SSTR2 during the differentiation of retinal organoid.** (A) The images of the process of establishment of retinal organoids. (B) The representative images of SSTR2 expression patterns at Day 45, Day 80, and Day 120.

**Figure S2. The transcriptional factors (TFs) upstream of *NRL* via SCENIC pipeline.** (A) Heatmap showing the TFs that positively correlated with *NRL* regulon activity revealed by SCENIC in the scRNA-seq dataset of human fetal retina. Top 25 TFs were highlighted in the box. Several TFs were identified as the putative TFs of *NRL*, *NR2E3* and *GNAT1*, the gene markers of rod photoreceptor.

**Figure S3. The increases of rod photoreceptors in the treatment of long-term somatostatin** (A) The Immunofluorescence staining of RXRG and PDE6H showing decreased numbers of cone photoreceptors. Scale bar, 50μm. (B) The immunofluorescence image showing significant upregulation of NR2E3 and RECOVERIN in the retinal organoids exposing to somatostatin from D80 until D130. The quantitative statistical analysis between control and somatostatin groups. (Scale bar, 50μm. **, p<0.01, n=6)

**Figure S4. The transduction of shSST showed co-expression of ISLET1 and mCherry** (A) The expression of *SST* and *ISLET1* in the scRNA-seq dataset of human retinal organoids. The western blotting showing the expression of SSTR2 and somatostatin. (B) The immunostaining of Day 80 retinal organoids showing the expression of SST in the inner layer (C) Co-staining of mCherry and RGC marker ISLET1 in shSST treatment at Day 94. The image of optical microscope showing strong mCherry expression at Day 94 (Scale bar, 200μm, n=6). (D) The CYN 154806 treatment increased the effect of *SST* knockdown in the retinal organoids (Scale bar, 50μm. *, p<0.05, n=6).

**Figure S5. SSTR2 signaling regulated pAKT expression** (A) Representative examples of pAKT immunoreactivity (red) showing an increase and cytoplasmic pattern in CYN 154806 treatment and decrease in somatostatin (SST) treatment. (Scale bar, 50μm. N=6).
